# Influence of impacted mandibular third molars on pathological changes on themselves and adjacent tissues across different age groups: a cross-sectional study

**DOI:** 10.1590/1678-7765-2025-0677

**Published:** 2026-03-30

**Authors:** Xiaoyue Ge, Yixiao Cui, Ning Li, Tianyang Lv, Yuntong Liang, Haochen Wu, Yuxuan Gu, Meiting Li, Changkui Liu, Yayuan Guo, Kaijin Hu

**Affiliations:** 1 Xi'an Medical University School of Stomatology Engineering Research Center of Oral and Maxillary System Disease Xi'an China Xi'an Medical University, School of Stomatology, Engineering Research Center of Oral and Maxillary System Disease, Xi'an, China.; 2 The Third Affiliated Hospital of Xi'an Medical University Xi'an China The Third Affiliated Hospital of Xi'an Medical University, Xi'an, China.

**Keywords:** Third molar, Alveolar bone loss, Cone-beam computed tomography

## Abstract

**Objective:**

To investigate the effects of different types of impacted mandibular third molar (IMTM) on lesions in situ and on adjacent tissues at different ages.

**Methodology:**

Cone Beam Computed Tomography (CBCT) images of 1007 IMTM cases were retrospectively analyzed. Associations between age, gender, impaction type, distal alveolar bone condition of mandibular second molar (MSM), IMTM-MSM contact status and occurrence of lesions on IMTM and its adjacent tissues were evaluated using Pearson’s chi-squared test and Fisher’s exact test.

**Results:**

MSM distal alveolar bone defects showed the highest incidence among all IMTM-related lesions (79.2%), followed by MSM distal external root resorption (ERR) (25.5%) and MSM distal caries (10.4%). Mesioangular (96.2%, p<0.001) and horizontal (95.9%, p<0.001) IMTM were associated with more severe bone defects and the incidence increased with age. IMTM-MSM surface-to-surface contact was significantly associated with MSM distal caries severity (12.5%, p<0.001) and distal ERR (29.5%, p<0.001). Inflammatory resorption of MSM distal alveolar bone was significantly correlated with MSM distal caries (47.1%, p<0.001), ERR (36.5%, p<0.05), distal alveolar bone defects (100%, p<0.001), and IMTM caries (14.1%, p<0.05). Study design followed the STROBE guidelines.

**Conclusion:**

Mesioangular impaction, horizontal impaction, MSM-IMTM surface-to-surface contact, and MSM distal alveolar bone in a state of inflammatory resorption greatly affect lesions on IMTM and its surrounding tissues. These findings suggest the prophylactic extraction in young adults (18–25 years) with high-risk impactions, while recommending observation or cautious management for asymptomatic older patients without signs of inflammatory bone resorption.

## Graphical abstract

**Figure:**
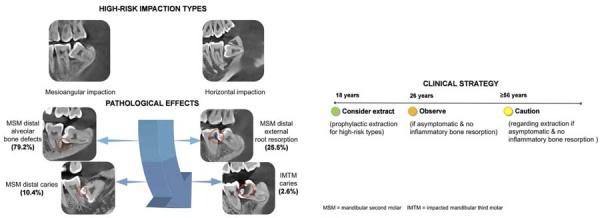

## Introduction

Impacted mandibular third molar (IMTM) extraction is generally indicated when a tooth has caused pathological changes in itself or surrounding tissues, or is likely to do so in the future. In contrast, the prophylactic extraction of asymptomatic, disease-free IMTMs remains controversial.^1-4^ Experts like Butzin contend that postponing extraction elevates the risk of complications, including cyst formation, periodontal disease, and resorptive damage to adjacent teeth, while also increasing the technical difficulty of future surgical procedures and diminishing patients’ quality of life.^1^ Others caution that many asymptomatic IMTMs may remain harmless throughout life and emphasize the potential risks of prophylactic extraction such as postoperative pain, swelling, infection, nerve injury, and mandibular fracture.^5^ Age is a critical factor, as older individuals are more susceptible to postoperative complications, particularly persistent pain and inferior alveolar nerve injury.^2^ Marqués et al. concluded that decisions regarding prophylactic extraction should primarily consider the risk of future pathological changes—such as cyst formation, pericoronitis, and caries or resorption in adjacent teeth—rather than demographic factors alone.^3^ Consequently, predicting the risk that an impacted tooth will lead to these pathologies represents a major clinical concern.

In earlier years, researchers often used methods such as root tip X-rays and panoramic X-rays to explore the effects of different IMTM locations on surrounding tissue lesions.^6-18^ Technological development, however, has established that Cone Beam Computed Tomography (CBCT) has advantages over two-dimensional X-rays in root resorption and caries detection, including a more reliable determination of alveolar bone defects and their severity than panoramic radiographs.^19-24^ Thus, most scholars have started using CBCT in their studies, but these have included few types of lesions and relatively small study sample sizes.^19-21,25-31^ Distinct from previous CBCT research, our study provides a more comprehensive analysis of diverse pathological changes associated with IMTMs, incorporating a wider range of influencing factors. It specifically investigates the impact of IMTM-MSM contact status and MSM distal alveolar bone condition, providing a more integrated evaluation of IMTM-associated pathologies by simultaneously incorporating anatomical, contact-related, and biological inflammatory factors. Particularly, including the IMTM–MSM contact status and distal alveolar bone inflammatory conditions enables a more nuanced assessment of lesion risk beyond traditional impaction classifications.

Based on CBCT imaging data, this retrospective analysis investigated how different IMTM types affect both the teeth and its surrounding tissues across various age groups, characterizing the patterns of associated lesion development. It further evaluated the lesion risks posed by different IMTM categories. Given its comprehensive data inclusion criteria, diverse types of lesions, and strict implementation plan, this study provides a theoretical basis and clinical reference for determining the timing of preventive IMTM removal.

## Methodology

### Study design

A multi-center, cross-sectional study was conducted from January 2024 to January 2025. CBCT images of 564 patients (1007 IMTMs, throughout 2024) were randomly collected from three independent medical units: a public specialty hospital, the dental department of a public general hospital, and a private dental hospital in Xi’an City, China. Our study was approved by the Ethics Committee of Xi’an Medical College (approval number: 2025YF-44, 2025JC-YBQN-1274).

Participants were adults aged 18 years and older who presented an impacted mandibular third molar with the adjacent second molar still present, and whose CBCT scans were clear and free of artifacts. All individuals were of Han ethnicity. Those with missing or severely damaged MSM and IMTM, patients with head and neck tumors, a history of mandibular surgery, and those with systemic diseases that severely affect the oral cavity were excluded.

All image evaluations were performed by an experienced calibrated operator (engaged in oral clinical work for over 20 years). The assessment methodology was standardized as detailed in Section *Variable assessment and definitions*.

### CBCT image acquisition and processing

The CBCT scans employed for imaging were acquired using an HDX WILL DENTRI-S CBCT unit (HDX WILL Co., Ltd., Seoul, South Korea). Exposure parameters were set at 85 kVp, 7.0 mA, with a field of view (FOV) of 16×14.5 cm and a voxel size of 200 μm. All the scans were completed by experienced operators from each institution following standardized procedures.

Tooth position was determined in the cross-sectional image by intersecting the coronal and sagittal lines perpendicularly at the MSM midpoint, while aligning the sagittal line through the midpoint of the first molar. In the sagittal image, the cross-sectional line was localized to the MSM cementoenamel junction (CEJ) and the coronal line was adjusted to coincide with the long axis of the MSM tooth. The sagittal line was adjusted to coincide with the long axis of the MSM in the coronal image.

Contact between the IMTM and MSM was assessed by Contact Status (categorized as no contact, point contact, or surface-to-surface contact). Type of impaction and impaction type were determined using two established classification systems. One, Winter’s classification, which classifies impaction based on the angle formed between the long axis of the adjacent tooth and the long axis of the impacted tooth: vertical impaction (–10° to 10°), mesioangular impaction (11° to 80°), horizontal impaction (81° to 100°), distoangular impaction (–11° to –80°), and other types of impaction (including inverted impaction, lingual impaction, buccal impaction);^7,8^ Two, Pell & Gregory’s classification:^32^ ①based on the relationship between the highest IMTM point and the occlusal plane of the adjacent MSM: Position A: the highest IMTM point aligns with or is above the occlusal plane of the adjacent MSM; Position B: the highest IMTM point is located between the neck of the adjacent MSM and its occlusal plane; Position C: the IMTM highest point is below the CEJ of the adjacent MSM. ②Based on the relationship between the distance from the distal aspect of the MSM to the mandibular ramus and the mesiodistal diameter of the IMTM: Class I: IMTM mesio-distal diameter is entirely located anterior to the anterior margin of the mandibular ramus; Class II: less than half of the IMTM mesio-distal diameter is located within the mandibular ramus; Class III: over half of the IMTM mesio-distal diameter is located within the mandibular ramus.

### Variable assessment and definitions

**Figure 1:**
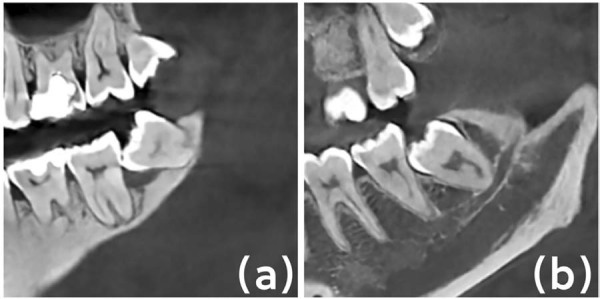
Health status of the MSM distal alveolar bone: (a) Inflammatory resorption; (b) Compressive resorption.

MSM distal alveolar bone condition: to further investigate the causes of MSM distal alveolar bone defects, MSM distal alveolar bone condition was classified into compressive resorption and inflammatory resorption. Compressive resorption primarily refers to alveolar bone defects caused by the spatial pressure exerted by the IMTM, whereas inflammatory resorption reflects the presence of pathological changes (Figure 1).

**Figure 2:**
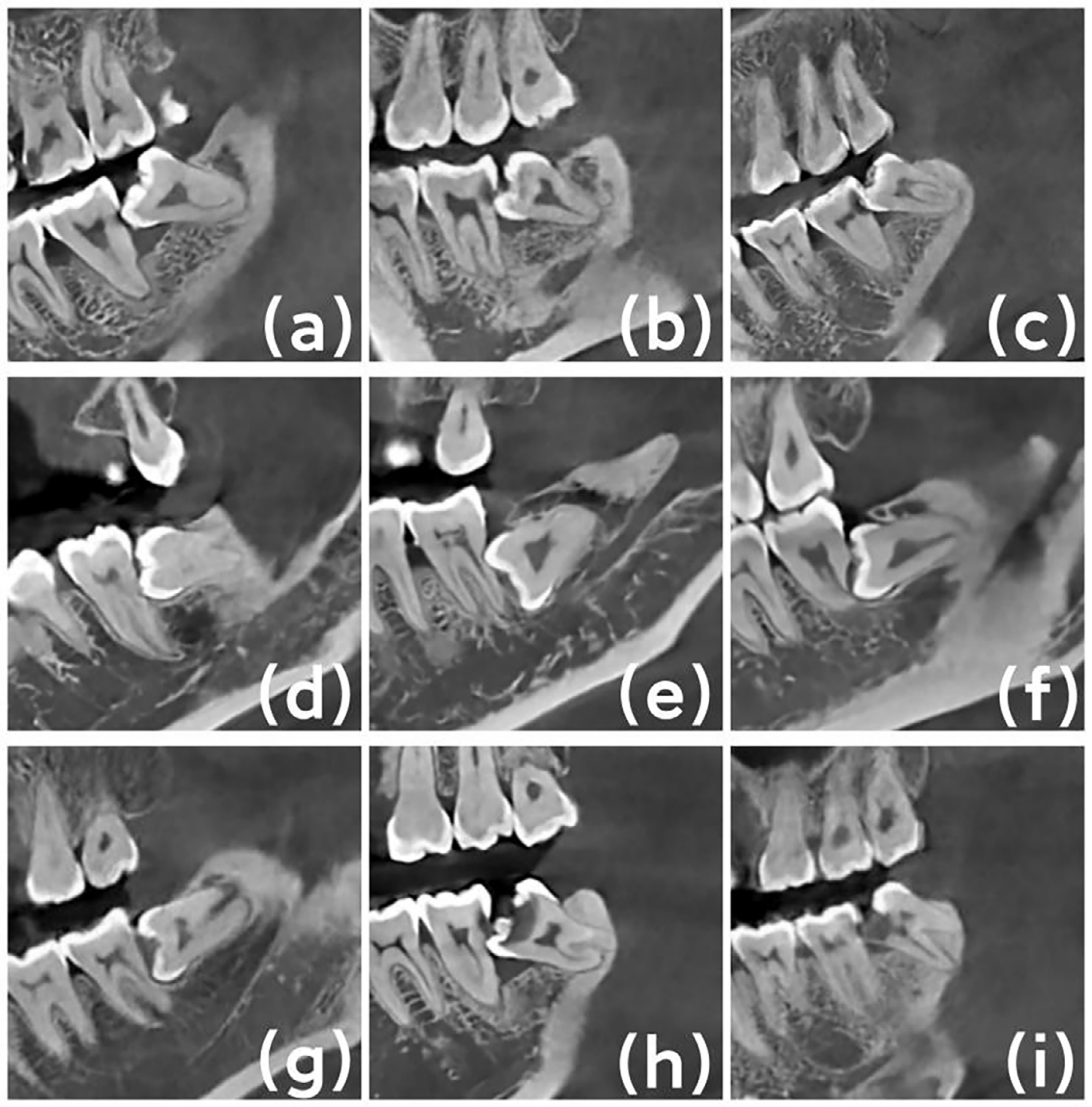
Severity of MSM distal caries: (a) Mild caries; (b) Moderate caries; (c) Severe caries. Severity of MSM distal root resorption: (d) Mild root resorption; (e) Moderate root resorption; (f) Severe root resorption. Severity of IMTM caries: (g) Mild caries; (h) Moderate caries; (i) Severe caries.

Severity of IMTM caries, MSM distal caries, and MSM external root resorption (ERR) was graded based on the relationship of the defects with the dentin and pulpal cavities.^19-21,33^ Mild: defects less than half the thickness of the dentin; Moderate: defects at least half the thickness of the dentin but not invading the pulp; Severe: defects invading the pulp chamber (Figure 2).

**Figure 3:**
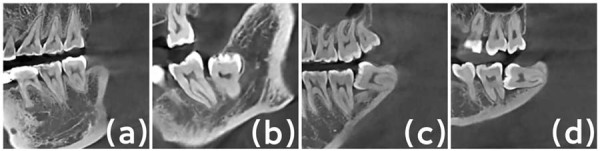
Severity of MSM alveolar bone defects: (a) No alveolar bone defect; (b) Mild alveolar bone defect; (c) Moderate alveolar bone defect; (d) Severe alveolar bone defect.

Severity of MSM alveolar bone defects were measured in the sagittal plane: distance from the MSM enamel-cementum junction to the level of the distal alveolar ridge (bone defect depth), and distance from the MSM enamel-cementum junction to the distal root apex (root length). Ratio was calculated by subtracting 2 mm from the measured bone defect depth and dividing it by the root length minus 2 mm. Based on the ratio, defect severity was classified according to the periodontal disease classification standards from the 1999 International Symposium^34^:(a) no alveolar bone defect: alveolar bone defect depth ≤ 2 mm; (b) mild alveolar bone defect: defect depth > 2 mm, ratio does not exceed 1/3; (c) moderate alveolar bone defect, ratio > 1/3, < 2/3; (d) severe alveolar bone defect, ratio ≥ 2/3 (Figure 3).

IMTM follicular spaces were classified by measuring the maximum diameter of the radiolucent area around the IMTM and grading the measurement results as less than 2 mm, 2 mm to 3 mm, or greater than 3 mm. Any cystic changes greater than 3 mm around the IMTM were considered pathological lesions (including odontogenic cysts or IMTM-associated tumors, etc.).^35,36^

### Data analysis

Measurement data were analyzed and processed on IBM SPSS Statistics 25.0. Continuous variables were verified for normality using Shapiro-Wilk’s test. Normally distributed variables were presented as mean ± standard deviation (SD). Categorical data were presented as frequencies and percentages. Continuous variable ‘age’ was converted into an ordinal categorical variable (18–25, 26–35, 36–45, 46–55, and ≥56 years) for analysis.

Reproducibility of the image measurements were endured by assessing intra-examiner reliability, having the primary operator re-measure a randomly selected 20% subsample of the IMTMs two weeks after the initial assessment. Measurement consistency was evaluated using the Weighted Kappa statistic for categorical variables. Additionally, two young dental workers with less than 5 years of working experience reviewed the 236 CBCT images and classified them. Consistency of their interpretations of the results was determined by calculating the Kappa value.

Univariate associations between IMTM-related lesions and demographic or imaging variables were analyzed by Pearson’s chi-squared test of independence and Fisher’s test. To control for potential confounders, all candidate variables were entered into a multivariate logistic regression model. Variable selection was performed using a backward stepwise elimination procedure. Prior to the regression analysis, collinearity diagnosis was performed to ensure that the variance inflation factors (VIF) were < 5, indicating no multicollinearity. Hosmer-Lemeshow’s test assessed the goodness-of-fit of the model (*p* > 0.05). These analyses were also used to perform subgroup comparisons to explore factors associated with each outcome. Results were considered statistically significant if p < 0.05.

## Results

### Descriptive statistical analysis of experimental data

Our study sample included 564 cases (1007 IMTM), 258 males (466 IMTM, 46.3%) and 306 females (541 IMTM, 53.7%), with mean age 36.65 ± 12.928 years. Of them, 118 cases (225 IMTM, 22.3%) were 18–25 years old, 163 cases (302 IMTM, 30.0%) were 26–35 years old, 142 cases (249 IMTM, 24.7%) were 36–45 years, 71 cases (118 IMTM, 11.7%) were 46–55 years, and 70 cases (113 IMTM, 11.2%) were ≥56 years (Table 1).

**Table 1:** Descriptive statistics of the study variables.

Variables	Number	Percentage
**Gender**		
Male (M)	466	46.3%
Female (F)	541	53.7%
**Age (years)**		
18–25	225(M:78 F:147)	22.3%
26–35	302(M:136 F:166)	30.0%
36–45	249(M:125 F:124)	24.7%
46–55	118(M:67 F:51)	11.7%
≥56	113(M:60 F:53)	11.2%
**Contact status between IMTM and MSM**		
No contact	155	15.4%
Point contact	36	3.6%
Surface-to-surface contact	816	81.0%
**MSM distal alveolar bone condition**		
Compressive resorption	922	91.6%
Inflammatory resorption	85	8.4%
**Winter's classification**		
Vertical impaction	376	37.3%
Mesioangular impaction	293	29.1%
Horizontal impaction	241	23.9%
Distoangular impaction	65	6.5%
Other types of impaction	32	3.2%
**Pell & Gregory’s classification (Occlusal)**		
Position A	529	52.5%
Position B	334	33.2%
Position C	144	14.3%
**Pell & Gregory’s classification (Ramus)**		
Class I	379	37.6%
Class II	450	44.7%
Class III	178	17.7%

### Association between MSM distal alveolar bone condition and its related influence factors

Different age groups were significantly associated with MSM distal alveolar bone condition, with 26–35-year-olds (*p*<0.001) presenting the highest incidence of MSM distal bone inflammatory resorption. Mesioangular impaction by Winter’s classification was significantly related with MSM distal bone inflammatory resorption (*p* < 0.001) (Table 2).

**Table 2:** Association between MSM distal alveolar bone condition and its related influencing factors.

	MSM distal alveolar bone condition		Total	*χ*^2^	*p*
	compressive resorption	inflammatory resorption			
	(N=922)	(N=85)			
					
**Age**					
18–25	221	4	225	23.120	0.000***
	(98.2%)	(1.8%)	(100.0%)		
26–35	263	39	302		
	(87.1%)	(12.9%)	(100.0%)		
36–45	224	25	249		
	(90.0%)	(10.0%)	(100.0%)		
46–55	107	11	118		
	(90.7%)	(9.3%)	(100.0%)		
≥56	107	6	113		
	(94.7%)	(5.3%)	(100.0%)		
**Winter’s classification**					
Vertical impaction	376	0	376	100.294	0.000***
	(100.0%)	(0.0%)	(100.0%)		
Mesioangular impaction	232	61	293		
	(79.2%)	(20.8%)	(100.0%)		
Horizontal impaction	218	23	241		
	(90.5%)	(9.5%)	(100.0%)		
Distoangular impaction	65	0	65		
	(100.0%)	(0.0%)	(100.0%)		
Other types of impaction	31	1	32		
	(96.9%)	(3.1%)	(100.0%)		

***p<0.001, indicating a statistically significant difference.

### Severity of MSM distal caries and its association with related influence factors

MSM distal caries showed an incidence of 10.4%, predominantly mild caries (6.9%). Mesioangular impaction (21.2%), Position A (16.1%), MSM-IMTM surface-to-surface contact (12.5%) and MSM distal alveolar bone inflammatory resorption (47.1%) were significantly associated with the occurrence of MSM distal caries (*p* < 0.001) (Table 3).

**Table 3:** Severity of MSM distal caries and its association with related influence factors.

Variables	Severity of MSM distal caries				Total	*χ*^2^	*p*
	No	Mild	Moderate	Severe	(N=1007)		
	(N=903)	(N=69)	(N=25)	(N=10)			
**Gender**							
Male	410	36	13	7	466	3.835	0.280
	(88.0%)	(7.7%)	(2.8%)	(1.5%)	(100.0%)		
Female	493	33	12	3	541		
	(91.1%)	(6.1%)	(2.2%)	(0.6%)	(100.0%)		
**Age**							
18–25	207	15	3	0	225	N/A^†^	0.160^†^
	(92.0%)	(6.7%)	(1.3%)	(0.0%)	(100.0%)		
26–35	265	24	11	2	302		
	(87.7%)	(7.9%)	(3.6%)	(0.7%)	(100.0%)		
36–45	219	19	8	3	249		
	(88.0%)	(7.6%)	(3.2%)	(1.2%)	(100.0%)		
46–55	111	3	1	3	118		
	(94.1%)	(2.5%)	(0.8%)	(2.5%)	(100.0%)		
≥56	101	8	2	2	113		
	(89.4%)	(7.1%)	(1.8%)	(1.8%)	(100.0%)		
**Winter’s classification**							
Vertical impaction	351	21	2	2	376	N/A^†^	0.000***^†^
	(93.4%)	(5.6%)	(0.5%)	(0.5%)	(100.0%)		
Mesioangular impaction	231	37	18	7	293		
	(78.8%)	(12.6%)	(6.1%)	(2.4%)	(100.0%)		
Horizontal impaction	228	7	5	1	241		
	(94.6%)	(2.9%)	(2.1%)	(0.4%)	(100.0%)		
Distoangular impaction	61	4	0	0	65		
	(93.8%)	(6.2%)	(0.0%)	(0.0%)	(100.0%)		
Other types of impaction	32	0	0	0	32		
	(100.0%)	(0.0%)	(0.0%)	(0.0%)	(100.0%)		
**Pell & Gregory’s classification (Ramus)**							
Class I	331	32	12	4	379	N/A^†^	0.071^†^
	(87.3%)	(8.4%)	(3.2%)	(1.1%)	(100.0%)		
Class II	401	32	11	6	450		
	(89.1%)	(7.1%)	(2.4%)	(1.3%)	(100.0%)		
Class III	171	5	2	0	178		
	(96.1%)	(2.8%)	(1.1%)	(0.0%)	(100.0%)		
**Pell & Gregory’s classification (Occlusal)**							
Position A	444	56	20	9	529	N/A^†^	0.000***^†^
	(83.9%)	(10.6%)	(3.8%)	(1.7%)	(100.0%)		
Position B	317	12	4	1	334		
	(94.9%)	(3.6%)	(1.2%)	(0.3%)	(100.0%)		
Position C	142	1	1	0	144		
	(98.6%)	(0.7%)	(0.7%)	(0.0%)	(100.0%)		
**Contact status between IMTM and MSM**							
No contact	154	1	0	0	155	N/A^†^	0.000***^†^
	(99.4%)	(0.6%)	(0.0%)	(0.0%)	(100.0%)		
Point contact	35	1	0	0	36		
	(97.2%)	(2.8%)	(0.0%)	(0.0%)	(100.0%)		
Surface-to-surface contact	714	67	25	10	816		
	(87.5%)	(8.2%)	(3.1%)	(1.2%)	(100.0%)		
**MSM distal alveolar bone condition**							
Compressive resorption	858	47	12	5	922	N/A^†^	0.000***^†^
	(93.1%)	(5.1%)	(1.3%)	(0.5%)	(100.0%)		
Inflammatory resorption	45	22	13	5	85		
	(52.9%)	(25.9%)	(15.3%)	(5.9%)	(100.0%)		

***p<0.001, indicating a statistically significant difference;

^†^*p*-values were calculated using Fisher’s exact test with Monte Carlo simulation (10,000 replications), as the chi-squared test was not applicable due to violations of its assumptions (≥20% of cells had expected counts of 1 < T < 5).

Variables that showed clinical significance or statistical significance in the univariate analysis were included in a multivariate logistic regression model. Results indicated surface-to-surface contact between MSM and IMTM as the strongest independent predictor, with an odds ratio (*OR*) of 10.023 (95% *CI*: 1.334–75.303, *p*=0.025). Inflammatory resorption of the MSM distal alveolar bone (*OR*=4.317, 95% *CI*: 2.298–8.109, *p*<0.001) and mesioangular impaction (*OR*=3.864, 95% *CI*: 2.083–7.168, *p*<0.001) also emerged as significant risk factors. Conversely, compared with Position A, Position B (*OR*=0.329) and Position C (*OR*=0.119) were associated with a significantly lower risk of distal caries (*p*<0.01). Hosmer-Lemeshow’s test confirmed a good model fit (*p*>0.05) (Table 4).

**Table 4:** Multivariate Logistic regression analysis of influence factors on MSM distal caries incidence.

Variables	*B*	*SE*	*OR*	*OR* (95%*CI*)		*p*
				Lower	Upper	
**Winter’s classification**						
Vertical position			1.000			0.000***
Mesioangular position	1.352	0.315	3.864	2.083	7.168	0.000***
Horizontal position	0.540	0.438	1.715	0.727	4.046	0.218
Distoangular position	0.055	0.561	1.057	0.352	3.176	0.921
Other types of position	-17.159	6.721.411	0.000	0.000		0.998
**Pell & Gregory’s classification (Ramus)**						
Position A			1.000			0.000***
Position B	-1.112	0.328	0.329	0.173	0.626	0.001**
Position C	-2.127	0.766	0.119	0.027	0.535	0.005**
**Contact status between IMTM and MSM**						
No contact			1.000			0.038*
Point contact	0.970	1.458	2.638	0.151	45.957	0.506
Surface-to-surface contact	2.305	1.029	10.023	1.334	75.303	0.025*
**MSM distal alveolar bone condition**						
Compressive resorption			1.000			
Inflammatory resorption	1.463	0.322	4.317	2.298	8.109	0.000***

*p<0.05, **p<0.01, ***p<0.001, indicating a statistically significant difference.

### Severity of MSM distal ERR and its association with related influence factors

MSM distal ERR showed a prevalence of 25.5%, predominantly mild (19.1%). Individuals aged 18–25 years exhibited the highest ERR prevalence (36.4%, *p*<0.001). Other types of impaction (46.9%), Position C (50.0%) and Class III (46.6%) were significantly associated with ERR occurrence (*p*<0.001). MSM-IMTM surface-to-surface contact yielded the highest ERR incidence (29.5%, *p* < 0.001). MSM distal alveolar bone inflammatory resorption significantly correlated with the occurrence of MSM distal ERR (36.5%, *p* < 0.05) (Table 5).

**Table 5:** Severity of MSM distal ERR and its association with related influence factors.

Variables	Severity of MSM distal ERR				Total	*χ*^2^	*p*
	No	Mild	Moderate	Severe	(N=1007)		
	(N=751)	(N=192)	(N=47)	(N=17)			
**Gender**							
Male	346	85	25	10	466	2.304	0.512
	(74.2%)	(18.2%)	(5.4%)	(2.1%)	(100.0%)		
Female	405	107	22	7	541		
	(74.9%)	(19.8%)	(4.1%)	(1.3%)	(100.0%)		
**Age**							
18–25	143	72	10	0	225	N/A^†^	0.000***^†^
	(63.6%)	(32.0%)	(4.4%)	(0.0%)	(100.0%)		
26–35	216	66	12	8	302		
	(71.5%)	(21.9%)	(4.0%)	(2.6%)	(100.0%)		
36–45	208	25	9	7	249		
	(83.5%)	(10.0%)	(3.6%)	(2.8%)	(100.0%)		
46–55	89	17	10	2	118		
	(75.4%)	(14.4%)	(8.5%)	(1.7%)	(100.0%)		
≥56	95	12	6	0	113		
	(84.1%)	(10.6%)	(5.3%)	(0.0%)	(100.0%)		
**Winter’s classification**							
Vertical impaction	369	7	0	0	376	N/A^†^	0.000***^†^
	(98.1%)	(1.9%)	(0.0%)	(0.0%)	(100.0%)		
Mesioangular impaction	166	95	21	11	293		
	(56.7%)	(32.4%)	(7.2%)	(3.8%)	(100.0%)		
Horizontal impaction	134	82	21	4	241		
	(55.6%)	(34.0%)	(8.7%)	(1.7%)	(100.0%)		
Distoangular impaction	65	0	0	0	65		
	(100.0%)	(0.0%)	(0.0%)	(0.0%)	(100.0%)		
Other types of impaction	17	8	5	2	32		
	(53.1%)	(25.0%)	(15.6%)	(6.3%)	(100.0%)		
**Pell & Gregory’s classification (Ramus)**							
Class I	353	20	4	2	379	125.571	0.000***^†^
	(93.1%)	(5.3%)	(1.1%)	(0.5%)	(100.0%)		
Class II	303	113	26	8	450		
	(67.3%)	(25.1%)	(5.8%)	(1.8%)	(100.0%)		
Class III	95	59	17	7	178		
	(53.4%)	(33.1%)	(9.6%)	(3.9%)	(100.0%)		
**Pell & Gregory’s classification (Occlusal)**							
Position A	466	52	10	1	529	126.974	0.000***
	(88.1%)	(9.8%)	(1.9%)	(0.2%)	(100.0%)		
Position B	213	93	19	9	334		
	(63.8%)	(27.8%)	(5.7%)	(2.7%)	(100.0%)		
Position C	72	47	18	7	144		
	(50.0%)	(32.6%)	(12.5%)	(4.9%)	(100.0%)		
**Contact status between IMTM and MSM**							
No contact	150	5	0	0	155	N/A^†^	0.000***^†^
	(96.8%)	(3.2%)	(0.0%)	(0.0%)	(100.0%)		
Point contact	26	10	0	0	36		
	(72.2%)	(27.8%)	(0.0%)	(0.0%)	(100.0%)		
Surface-to-surface contact	575	177	47	17	816		
	(70.5%)	(21.7%)	(5.8%)	(2.1%)	(100.0%)		
**MSM distal alveolar bone condition**							
Compressive resorption	697	167	41	17	922	N/A^†^	0.028*^†^
	(75.6%)	(18.1%)	(4.4%)	(1.8%)	(100.0%)		
Inflammatory resorption	54	25	6	0	85		
	(63.5%)	(29.4%)	(7.1%)	(0.0%)	(100.0%)		

* *p*<0.05, *** *p*<0.001

^†^ *p*-values were calculated using Fisher’s exact test with Monte Carlo simulation (10,000 replications), as the chi-squared test was not applicable due to violations of its assumptions (≥20% of cells had expected counts of 1 < T < 5).

Multivariate logistic regression identified the independent predictors of MSM distal ERR. Analysis revealed mesioangular impaction as the strongest independent risk factor (*OR*=25.263, 95% *CI*: 11.283–56.566, *p*<0.001) compared with vertical impaction. Other significant anatomical risk factors included horizontal impaction (*OR*=20.351), other impaction types (*OR*=23.294), Pell & Gregory’s Level C (*OR*=2.734), and Class II/III positions (*p*<0.05).

Regarding age, the model indicated that compared with the 18–25 age group, the risk of ERR was significantly lower in the 36–45 age group (*OR*=0.470, *p* = 0.005) and the ≥56 age group (*OR*=0.468, *p*=0.040), suggesting that younger age is an independent risk factor for ERR. Hosmer-Lemeshow’s test indicated a satisfactory model fit (p>0.05) (Table 6).

**Table 6:** Multivariate Logistic regression analysis of influence factors on the incidence of MSM distal ERR.

Variables	*B*	*SE*	*OR*	*OR*(95%*CI*)		*p*
				Lower	Upper	
**Age**						
18-25			1.000			0.033*
26-35	-0.197	0.219	0.821	0.535	1.261	0.368
36-45	-0.756	0.268	0.470	0.278	0.794	0.005**
46-55	-0.124	0.331	0.883	0.462	1.689	0.707
≥56	-0.758	0.370	0.468	0.227	0.968	0.040*
**Winter’s classification**						
Vertical position			1.000			0.000***
Mesioangular position	3.229	0.411	25.263	11.283	56.566	0.000***
Horizontal position	3.013	0.428	20.351	8.798	47.076	0.000***
Distoangular position	-17.403	4.896.473	0.000	0.000	.	0.997
Other types of position	3.148	0.549	23.294	7.935	68.380	0.000***
**Pell & Gregory’s classification (Ramus)**						
Class I			1.000			0.079
Class II	0.589	0.275	1.802	1.050	3.091	0.033*
Class III	0.676	0.328	1.967	1.035	3.738	0.039*
**Pell & Gregory’s classification (Occlusal)**						
Position A			1.000			0.002**
Position B	0.317	0.215	1.373	0.900	2.095	0.141
Position C	1.006	0.291	2.734	1.546	4.836	0.001**

*p<0.05, **p<0.01, ***p<0.001, indicating a statistically significant difference.

### Severity of IMTM caries and its association with related influence factors

Incidence of IMTM caries was 2.6%, predominantly mild severity (1.9%), with the highest incidence (14.2%) found in those ≥56 years of age (*p*<0.01). Class I (11.3%, *p*<0.001) and Position A (9.6%, *p*<0.01) were significantly associated with IMTM caries. Incidence of IMTM caries showed a significant difference in the presence of MSM distal alveolar bone inflammatory resorption in relation to IMTM caries severity (14.1%, *p*<0.05) (Table 7).

**Table 7:** Severity of IMTM caries and its association with related influence factors.

Variables	Severity of IMTM caries				Total	*χ*^2^	*p*
	No	Mild	Moderate	Severe	(N=1007)		
	(N=939)	(N=52)	(N=9)	(N=7)			
**Gender**							
Male	430	25	5	6	466	N/A^†^	0.188^†^
	(92.3%)	(5.4%)	(1.1%)	(1.3%)	(100.0%)		
Female	509	27	4	1	541		
	(94.1%)	(5.0%)	(0.7%)	(0.2%)	(100.0%)		
**Age**							
18–25	220	5	0	0	225	N/A^†^	0.007**^†^
	(97.8%)	(2.2%)	(0.0%)	(0.0%)	(100.0%)		
26–35	279	19	3	1	302		
	(92.4%)	(6.3%)	(1.0%)	(0.3%)	(100.0%)		
36–45	234	12	2	1	249		
	(94.0%)	(4.8%)	(0.8%)	(0.4%)	(100.0%)		
46–55	109	5	2	2	118		
	(92.4%)	(4.2%)	(1.7%)	(1.7%)	(100.0%)		
≥56	97	11	2	3	113		
	(85.8%)	(9.7%)	(1.8%)	(2.7%)	(100.0%)		
**Winter’s classification**							
Vertical impaction	345	24	4	3	376	N/A^†^	0.461^†^
	(91.8%)	(6.4%)	(1.1%)	(0.8%)	(100.0%)		
Mesioangular impaction	270	18	2	3	293		
	(92.2%)	(6.1%)	(0.7%)	(1.0%)	(100.0%)		
Horizontal impaction	232	7	1	1	241		
	(96.3%)	(2.9%)	(0.4%)	(0.4%)	(100.0%)		
Distoangular impaction	60	3	2	0	65		
	(92.3%)	(4.6%)	(3.1%)	(0.0%)	(100.0%)		
Other types of impaction	32	0	0	0	32		
	(100.0%)	(0.0%)	(0.0%)	(0.0%)	(100.0%)		
**Pell & Gregory’s classification (Ramus)**							
Class I	336	33	7	3	379	N/A^†^	0.000***^†^
	(88.7%)	(8.7%)	(1.8%)	(0.8%)	(100.0%)		
Class II	428	17	2	3	450		
	(95.1%)	(3.8%)	(0.4%)	(0.7%)	(100.0%)		
Class III	175	2	0	1	178		
	(98.3%)	(1.1%)	(0.0%)	(0.6%)	(100.0%)		
**Pell & Gregory’s classification (Occlusal)**							
Position A	478	39	8	4	529	N/A^†^	0.004**^†^
	(90.4%)	(7.4%)	(1.5%)	(0.8%)	(100.0%)		
Position B	322	10	1	1	334		
	(96.4%)	(3.0%)	(0.3%)	(0.3%)	(100.0%)		
Position C	139	3	0	2	144		
	(96.5%)	(2.1%)	(0.0%)	(1.4%)	(100.0%)		
**Contact status between IMTM and MSM**							
No contact	148	4	0	3	155	N/A^†^	0.207^†^
	(95.5%)	(2.6%)	(0.0%)	(1.9%)	(100.0%)		
Point contact	35	1	0	0	36		
	(97.2%)	(2.8%)	(0.0%)	(0.0%)	(100.0%)		
Surface-to-surface contact	756	47	9	4	816		
	(92.6%)	(5.8%)	(1.1%)	(0.5%)	(100.0%)		
**MSM distal alveolar bone condition**							
Compressive resorption	866	43	7	6	922	N/A^†^	0.026*^†^
	(93.9%)	(4.7%)	(0.8%)	(0.7%)	(100.0%)		
Inflammatory resorption	73	9	2	1	85		
	(85.9%)	(10.6%)	(2.4%)	(1.2%)	(100.0%)		

*p<0.05, **p<0.01, ***p<0.001

^†^*p*-values were calculated using Fisher’s exact test with Monte Carlo simulation (10,000 replications), as the chi-squared test was not applicable due to violations of its assumptions (≥20% of cells had expected counts of 1 < T < 5).

A multivariate logistic regression model was constructed using variables significant in the univariate analysis. Analysis showed that age ≥56 years was a significant independent risk factor (*OR*=4.240, 95% *CI*: 1.431–12.559, *p*=0.009) compared with the 18–25 age group. Inflammatory resorption of the MSM distal alveolar bone also significantly increased the risk of IMTM caries (*OR*=2.364, 95% *CI*: 1.180–4.734, *p*=0.015). Regarding the mandibular ramus relation, Class II (*OR*=0.474) and Class III (*OR*=0.164) positions were associated with a significantly lower risk of caries compared with Class I, suggesting that IMTMs with sufficient eruption space (Class I) are more susceptible to decay, likely due to their functional position and relative exposure to the oral environment (Table 8).

**Table 8:** Multivariate Logistic regression analysis of influence factors on the incidence of IMTM caries.

Variables	*B*	*SE*	*OR*	*OR*(95%*CI*)		*p*
				Lower	Upper	
**Age**						
18-25			1.000			0.059
26-35	0.988	0.513	2.687	0.983	7.344	0.054
36-45	0.576	0.544	1.780	0.613	5.168	0.289
46-55	0.886	0.589	2.425	0.765	7.687	0.132
≥56	1.445	0.554	4.240	1.431	12.559	0.009**
**Pell & Gregory’s classification (Ramus)**						
Class I			1.000			0.002**
Class II	-0.747	0.293	0.474	0.267	0.841	0.011*
Class III	-1.811	0.611	0.164	0.049	0.542	0.003**
**MSM distal alveolar bone condition**						
Compressive resorption			1.000			
Inflammatory resorption	0.860	0.354	2.364	1.180	4.734	0.015*

*p<0.05, **p<0.01, indicating a statistically significant difference.

### Severity of MSM distal bone defects and its association with related influence factors

MSM distal alveolar bone defects reached 79.2% and were predominantly mild (43.6%). Gender (*p*<0.01), age (*p*<0.001), impaction type (*p*<0.001), IMTM-MSM contact status (*p*<0.001) and MSM distal alveolar bone condition (*p*<0.001) were significantly associated with the severity of MSM distal bone defects. Incidence of alveolar bone defects increased with age. Mesioangular impaction exhibited the highest incidence of alveolar bone defects (96.2%, p<0.001).Position C (96.5%) and Class III (93.8%) were significantly associated with alveolar bone defects (*p*<0.001). IMTM-MSM contact status exhibited the highest incidence of alveolar bone defects (91.7%, *p*<0.001). Incidence of MSM distal alveolar bone defects nearly reached 100.0% in the case of inflammatory resorption (*p*<0.001) (Table 9).

**Table 9:** Severity of MSM distal alveolar bone defects and its association with related influence factors.

Variables	Severity of MSM distal alveolar bone defects				Total	*χ*^2^	*p*
	No	Mild	Moderate	Severe	(N=1007)		
	(N=209)	(N=439)	(N=248)	(N=111)			
**Gender**							
Male	80	191	139	56	466	17.036	0.001^*^
	(17.2%)	(41.0%)	(29.8%)	(12.0%)	(100.0%)		
Female	129	248	109	55	541		
	(23.8%)	(45.8%)	(20.1%)	(10.2%)	(100.0%)		
**Age**							
18–25	49	109	57	10	225	43.525	0.000***
	(21.8%)	(48.4%)	(25.3%)	(4.4%)	(100.0%)		
26–35	73	116	84	29	302		
	(24.2%)	(38.4%)	(27.8%)	(9.6%)	(100.0%)		
36–45	60	96	55	38	249		
	(24.1%)	(38.6%)	(22.1%)	(15.3%)	(100.0%)		
46–55	16	55	25	22	118		
	(13.6%)	(46.6%)	(21.2%)	(18.6%)	(100.0%)		
≥56	11	63	27	12	113		
	(9.7%)	(55.8%)	(23.9%)	(10.6%)	(100.0%)		
**Winter’s classification**							
Vertical impaction	146	206	22	2	376	463.018	0.000***
	(38.8%)	(54.8%)	(5.9%)	(0.5%)	(100.0%)		
Mesioangular impaction	11	150	100	32	293		
	(3.8%)	(51.2%)	(34.1%)	(10.9%)	(100.0%)		
Horizontal impaction	10	49	111	71	241		
	(4.1%)	(20.3%)	(46.1%)	(29.5%)	(100.0%)		
Distoangular impaction	37	25	2	1	65		
	(56.9%)	(38.5%)	(3.1%)	(1.5%)	(100.0%)		
Other types of impaction	5	9	13	5	32		
	(15.6%)	(28.1%)	(40.6%)	(15.6%)	(100.0%)		
**Pell & Gregory’s classification (Ramus)**							
Class I	135	189	38	17	379	206.124	0.000***
	(35.6%)	(49.9%)	(10.0%)	(4.5%)	(100.0%)		
Class II	63	198	148	41	450		
	(14.0%)	(44.0%)	(32.9%)	(9.1%)	(100.0%)		
Class III	11	52	62	53	178		
	(6.2%)	(29.2%)	(34.8%)	(29.8%)	(100.0%)		
**Pell & Gregory’s classification (Occlusal)**							
Position A	173	286	58	12	529	422.933	0.000***
	(32.7%)	(54.1%)	(11.0%)	(2.3%)	(100.0%)		
Position B	31	127	146	30	334		
	(9.3%)	(38.0%)	(43.7%)	(9.0%)	(100.0%)		
Position C	5	26	44	69	144		
	(3.5%)	(18.1%)	(30.6%)	(47.9%)	(100.0%)		
**Contact status between IMTM and MSM**							
No contact	16	58	54	27	155	34.068	0.000***
	(10.3%)	(37.4%)	(34.8%)	(17.4%)	(100.0%)		
Point contact	3	14	13	6	36		
	(8.3%)	(38.9%)	(36.1%)	(16.7%)	(100.0%)		
Surface-to-surface contact	190	367	181	78	816		
	(23.3%)	(45.0%)	(22.2%)	(9.6%)	(100.0%)		
**MSM distal alveolar bone condition**							
Compressive resorption	209	410	208	95	922	45.273	0.000***
	(22.7%)	(44.5%)	(22.6%)	(10.3%)	(100.0%)		
Inflammatory resorption	0	29	40	16	85		
	(0.0%)	(34.1%)	(47.1%)	(18.8%)	(100.0%)		

**p<0.01, ***p<0.001

Variables significant in the univariate analysis were included in a multivariate logistic regression model. Results indicated mesioangular impaction as a major independent risk factor, with an *OR* of 7.955 (95% *CI*: 4.083–15.499, *p*<0.001) compared with vertical impaction. Horizontal impaction (*OR*=5.582, *p*<0.001) also significantly increased the risk of bone defects. Regarding impaction depth, Position C (*OR*=6.577, *p*<0.001) and Position B (*OR*=2.603, *p*<0.001) emerged as significant influencing factors compared with Position A. Interestingly, distoangular position (*OR*=0.412, *p*=0.002) appeared to be a protective factor relative to vertical impaction. Although inflammatory resorption showed a very high *OR*, it served as a near-perfect predictor since 100% of such cases exhibited bone defects. Hosmer-Lemeshow’s test indicated a good model fit (p>0.05) (Table 10).

**Table 10:** Multivariate Logistic regression analysis of influence factors on the incidence of MSM distal alveolar bone defects.

Variables	*B*	*SE*	*OR*	*OR*(95%*C*I)		*p*
				Lower	Upper	
**Winter’s classification**						
Vertical position			1.000			0.000***
Mesioangular position	2.074	0.340	7.955	4.083	15.499	0.000***
Horizontal position	1.720	0.378	5.582	2.661	11.710	0.000***
Distoangular position	-0.887	0.284	0.412	0.236	0.718	0.002**
Other types of position	0.152	0.551	1.164	0.395	3.430	0.783
**Pell & Gregory’s classification (Occlusal)**						
Position A			1.000			0.000***
Position B	0.956	0.257	2.603	1.574	4.303	0.000***
Position C	1.884	0.510	6.577	2.421	17.864	0.000***
**MSM distal alveolar bone condition**						
Compressive resorption			1.000			
Inflammatory resorption	18.856	4.302.723	154.543.459.322	0.000	.	0.997

### Prevalence of IMTM follicular spaces and its association with related influence factors

Gender, age, impaction type, IMTM-MSM contact status and MSM distal alveolar bone condition were not statistically associated with occurrence of IMTM follicular spaces (*p*>0.05). Only distoangular impaction causing a 2mm–3mm IMTM follicular space (4.6%) and >3 mm cystic lesions (9.2%) were higher than the other impaction types (*p*<0.001) (Table 11).

**Table 11:** Prevalence of IMTM follicular spaces and its association with related influence factors.

Variables	prevalence of IMTM follicular spaces			Total	*χ*^2^	*p*
	<2mm (N=974)	2mm–3mm (N=14)	>3mm (N=19)	(N=1007)		
**Gender**						
Male	450	7	9	466	0.089	0.956
	(96.6%)	(1.5%)	(1.9%)	(100.0%)		
Female	524	7	10	541		
	(96.9%)	(1.3%)	(1.8%)	(100.0%)		
**Age**						
18–25	220	2	3	225	N/A^†^	0.626^†^
	(97.8%)	(0.9%)	(1.3%)	(100.0%)		
26–35	287	6	9	302		
	(95.0%)	(2.0%)	(3.0%)	(100.0%)		
36–45	239	5	5	249		
	(96.0%)	(2.0%)	(2.0%)	(100.0%)		
46–55	117	0	1	118		
	(99.2%)	(0.0%)	(0.8%)	(100.0%)		
≥56	111	1	1	113		
	(98.2%)	(0.9%)	(0.9%)	(100.0%)		
**Winter’s classification**						
Vertical impaction	362	6	8	376	N/A^†^	0.000***^†^
	(96.3%)	(1.6%)	(2.1%)	(100.0%)		
Mesioangular impaction	289	3	1	293		
	(98.6%)	(1.0%)	(0.3%)	(100.0%)		
Horizontal impaction	237	1	3	241		
	(98.3%)	(0.4%)	(1.2%)	(100.0%)		
Distoangular impaction	56	3	6	65		
	(86.2%)	(4.6%)	(9.2%)	(100.0%)		
Other types of impaction	30	1	1	32		
	(93.8%)	(3.1%)	(3.1%)	(100.0%)		
**Pell & Gregory’s classification (Ramus)**						
Class I	373	2	4	379	N/A^†^	0.130^†^
	(98.4%)	(0.5%)	(1.1%)	(100.0%)		
Class II	429	10	11	450		
	(95.3%)	(2.2%)	(2.4%)	(100.0%)		
Class III	172	2	4	178		
	(96.6%)	(1.1%)	(2.2%)	(100.0%)		
**Pell & Gregory’s classification (Occlusal)**						
Position A	506	9	14	529	N/A^†^	0.216^†^
	(95.7%)	(1.7%)	(2.6%)	(100.0%)		
Position B	326	5	3	334		
	(97.6%)	(1.5%)	(0.9%)	(100.0%)		
Position C	142	0	2	144		
	(98.6%)	(0.0%)	(1.4%)	(100.0%)		
**Contact status between IMTM and MSM**						
No contact	149	4	2	155	N/A^†^	0.524^†^
	(96.1%)	(2.6%)	(1.3%)	(100.0%)		
Point contact	35	0	1	36		
	(97.2%)	(0.0%)	(2.8%)	(100.0%)		
Surface-to-surface contact	790	10	16	816		
	(96.8%)	(1.2%)	(2.0%)	(100.0%)		
**MSM distal alveolar bone condition**						
Compressive resorption	890	14	18	922	N/A^†^	0.775^†^
	(96.5%)	(1.5%)	(2.0%)	(100.0%)		
Inflammatory resorption	84	0	1	85		
	(98.8%)	(0.0%)	(1.2%)	(100.0%)		

***p<0.001, indicating a statistically significant difference.

^†^*p*-values were calculated using Fisher’s exact test with Monte Carlo simulation (10,000 replications), as the chi-squared test was not applicable due to violations of its assumptions (≥20% of cells had expected counts of 1 < T < 5).

Finally, consistency analysis between the internal examiners and the inspectors using the method mentioned in Section 2.4 yielded a kappa value of 0.67, with *p*<0.01. Kappa value for the consistency test between external examiners was 0.71, with *p*<0.001.

## Discussion

Distal caries prevalence reached 10.4% in mandibular second molars (MSMs), differing from previous reports. Reported rates were 3.4% and 4.3% in the studies by Akkitap and Gumru^19^ (2023) and Abat, et al.^37^ (2025), whereas Ndiaye, et al.^12^ (2021) and Chen, et al.^20^ (2020) reported rates of 33% and 31.6%, respectively. These discrepancies may be attributed to variations in radiographic methods and sample characteristics: while Ndiaye, et al.^12^ (2021) utilized panoramic radiographs with a limited sample size (n=386), Akkitap and Gumru^19^ (2023) and Abat, et al.^37^ (2025) employed CBCT but included both maxillary and mandibular third molars, resulting in a broader scope. Data from Chen, et al.^20^ (2020) were derived from a specialized hospital. The present study selected samples from three different types of hospitals, enhancing representativeness. We confirmed a significant association between mesioangular impaction and distal caries in MSMs (*p* < 0.001), consistent with findings by Skitioui, et al.^7^ (2023) and Poszytek and Górski^8^ (2023). Position A was associated with a higher risk.^19^ Mesioangular and horizontal impactions are more prone to caries due to extensive CEJ exposure, which facilitates food impaction and complicates oral hygiene maintenance.^8^ Our multivariate analysis further confirmed that surface-to-surface contact (*OR* = 10.023) and inflammatory bone resorption (*OR* = 4.317) are the most powerful independent predictors for MSM distal caries, suggesting that the physical proximity and the biological inflammatory environment outweigh other anatomical factors in driving the decay process.

Prevalence of MSM ERR was 25.5%, falling within the range reported in previous studies (11.6%-33.4%).^7,9,11,19,25,27,28,37^ Studies utilizing panoramic radiography, such as Alqahtani, et al.^9^ (2025) and Haddad, et al.^11^ (2021), reported lower rates, suggesting that CBCT offers superior detection capability for ERR. Smaller sample sizes and single-center designs may have contributed to the higher rates reported in some CBCT studies, such as Akkitap and Gumru^19^ (2023), Sakhdari, et al.^27^ (2021), and Gürses, et al.^29^ (2023). Mesioangular, horizontal, and other impaction types were significantly associated with ERR (p < 0.001), which is in line with the results of a recent CBCT assessment.^37^ Class III and Position C impactions were associated with a higher risk of ERR, consistent with Cui et al.^28^ (2024). Prevalence of IMTMs caries was 2.6%, similar to the 4.2% reported by Yıldırım and Dindar (2022),^14^ but lower than the 18.9% reported by Altan and Akbulut^10^ (2019). This variation may stem from differences in sample sources (this study was multicenter) and caries inclusion criteria. In contrast to Altan and Akbulut^10^ (2019), we found no significant association was found between Winter’s classification and IMTM caries (*p* > 0.05), which may be related to the overall low caries incidence.

Prevalence of MSM distal alveolar bone defects was 79.2%, comparable to the 70.9% and 69.1% reported by Akkitap and Gumru^19^ (2023) and Abat, et al.^37^ (2025), respectively, but higher than the 18.21% reported by Poszytek and Górski^8^ (2023). These differences likely arise from methodological and diagnostic criteria variations: we utilized CBCT and defined a bone defect as depth >2 mm based on the 1999 International Workshop criteria,^34^ and further introduced a defect depth-to-root length ratio for grading, offering greater clinical systematicity. Mesioangular and horizontal impactions exhibited bone defect rates exceeding 95%, and Positions B and C were also identified as high-risk factors, consistent with previous reports.^7,10,19,37^ Prevalence of IMTM cystic changes was 1.9%, lower than rates reported by Altan and Akbulut^10^ (2019), Haddad, et al.^11^ (2021), and Akkitap and Gumru^19^ (2023). This discrepancy may be related to the diagnostic threshold applied (this study used a follicular width >3 mm) and imaging methodology. Rate of cystic changes is approximately only 2.20% when the follicular width is 2–3 mm.^35^ Distoangular impactions were more frequently associated with follicular spaces of 2–3 mm (4.6%) and >3 mm (9.2%), and a significant association was observed between Winter’s classification and follicular size (*p* < 0.001).^10^ We further investigated the influence of IMTM-MSM contact and MSM distal alveolar bone status on related pathologies. Surface-to-surface contact was significantly associated with MSM distal caries (12.5%) and ERR (29.5%) (*p* < 0.001), whereas point contact was associated with bone defects (91.7%, *p* < 0.001). Inflammatory resorption of the distal alveolar bone was also significantly associated with MSM distal caries (47.1%), ERR (36.5%), bone defects (100%), and IMTM caries (14.1%) (*p*<0.05). These findings provide new references for the clinical decision-making regarding prophylactic extraction of IMTMs.

Abat, et al.^37^ (2025) reported a positive correlation between age and caries prevalence and severity, external root resorption, and alveolar bone defects in second molars adjacent to mesioangular or horizontally IMTMs, further identifying age as a critical factor in developing these adjacent pathologies.^37,38^ Baeza, et al.^39^ (2021) reported that the likelihood of postoperative complications is 1.5 times higher in older patients than in those under 25, with extraction difficulty also increasing due to bone calcification, indicating that IMTM extraction is contraindicated in this population. Thus, we suggest prophylactic extraction for 18–25 years old as younger age was identified as an independent risk factor for MSM distal ERR (with risk significantly decreasing in the 36–45 and ≥56 groups (*OR* = 0.470 and 0.468, respectively). In this age group, the IMTM apices are not yet fully developed and still have growth momentum, which may cause complications in the future, especially when IMTM are located in the mesioangular and horizontal impaction that are prone to cause related lesions. Age ≥56 years should be more cautious about extraction choices if symptoms and MSM inflammatory resorption are lacking due to a fully developed root apex, no growth momentum and a higher likelihood of post-extraction complications and difficulty in extracting the tooth. A total of 1007 IMTM cases data were included here, which characterizes a large sample size compared with the previous relevant studies based on CBCT.^19-21,25-29,31^Some studies with sample sizes ranging from 115 to 421 IMTMs,^19-21,25,27-29,31^ and most of the articles only included one or more IMTM-associated lesions. For example, Tunç and Koc^25^ (2020) explored the risk factors associated with MSM caries and ERR; Chen, et al.^20^ (2020) only investigated the relation between IMTM and MSM distal caries, and some scholars only investigated the correlation between IMTM and MSM distal ERR.^21,27-30^ Kou, et al. (2025) had a larger sample size (1150 IMTMs) than the present study but only investigated one lesion—MSM distal ERR.^30^ To investigate whether asymptomatic and lesion-free IMTM should be extracted prophylactically, it is important to consider as many IMTM-related lesions as possible; thus, five lesions—MSM distal caries, MSM distal ERR, MSM distal alveolar bone defect, IMTM caries, and IMTM follicular spaces—were included in this study.

Although this study included a relatively large sample size, an even larger cohort may be required in future research due to the low incidence of certain specific lesions. Our analysis was based solely on CBCT imaging. While CBCT offers high-resolution images and superior spatial detail, it may not accurately detect some subtle lesions such as early-stage caries in pits and fissures. Thus, a combination of radiographic imaging and clinical examination would be more effective in accurately identifying such conditions. Additionally, the study population exclusively including Han ethnicity may limit the external validity of our findings. While the Han population is the largest ethnic group in China, potential variations in craniofacial morphology and dental arch characteristics among different ethnic groups could influence IMTM impaction patterns and associated pathologies. Moreover, considering selection bias, the limitations of geographical population differences, and economic disparities, future in-depth research should focus on China’s rural areas or collaborate with international cooperation to ensure that the research results are more applicable to the specific circumstances in China and even the global scenario.

## Conclusion

CBCT analysis on the effects of different IMTM types on IMTM and its surrounding tissue lesions showed that mesioangular impaction, horizontal impaction, MSM-IMTM surface-to-surface contact, and inflammatory resorption of the MSM distal alveolar bone greatly affect lesion on IMTM and its surrounding tissues. Given the cross-sectional study design, the following management suggestions should be considered with caution, as they derive from observed associations rather than longitudinal evidence of outcomes:(1) Prophylactic extraction may be considered for patients aged 18–25 years, especially when the IMTM type is prone to associated lesions such as mesioangular or horizontal impaction; (2) For patients aged 26–55 years without evidence of MSM distal bone inflammatory resorption or symptoms, a conservative, observational approach appears justifiable; (3) For patients aged ≥56 years under the same conditions, more caution should be exercised regarding extraction.
